# Transbronchial Lung Cryobiopsy in the Diagnosis of Fibrotic Interstitial Lung Diseases

**DOI:** 10.1371/journal.pone.0086716

**Published:** 2014-02-28

**Authors:** Gian Luca Casoni, Sara Tomassetti, Alberto Cavazza, Thomas V. Colby, Alessandra Dubini, Jay H. Ryu, Elisa Carretta, Paola Tantalocco, Sara Piciucchi, Claudia Ravaglia, Christian Gurioli, Micaela Romagnoli, Carlo Gurioli, Marco Chilosi, Venerino Poletti

**Affiliations:** 1 Department of Diseases of the Thorax, G.B Morgagni Hospital, Forlì, Italy; 2 Department of Pathology, S. Maria Nuova Hospital-I.R.C.C.S, Reggio Emilia, Italy; 3 Department of Pathology, Mayo Clinic, Scottsdale, Arizona, United States of America; 4 Department of Pathology, G.B Morgagni Hospital, Forlì, Italy; 5 Division of Pulmonary and Critical Care Medicine, Mayo Clinic, Rochester, Minnesota, United States of America; 6 Biostatistics and Clinical Trials Unit, Istituto Scientifico Romagnolo per lo Studio e la Cura dei Tumori, Meldola Forlì-Cesena, Italy; 7 Department of Radiology, G.B Morgagni Hospital, Forlì, Italy; 8 Department of Pathology, Verona University, Verona, Italy; McMaster University, Canada

## Abstract

**Background:**

Histology is a key element for the multidisciplinary diagnosis of fibrotic diffuse parenchymal lung diseases (f-DPLD) when the clinical-radiological picture is nondiagnostic. Transbronchial lung cryobiopsy (TBLC) have been shown to be useful for obtaining large and well-preserved biopsies of lung parenchyma, but experience with TBLC in f-DPLD is limited.

**Objectives:**

To evaluate safety, feasibility and diagnostic yield of TBLC in f-DPLD.

**Method:**

Prospective study of 69 cases of TBLC using flexible cryoprobe in the clinical-radiological setting of f-DPLD with nondiagnostic high resolution computed tomography (HRCT) features.

**Results:**

Safety: pneumothorax occurred in 19 patients (28%). One patient (1.4%) died of acute exacerbation. Feasibility: adequate cryobiopsies were obtained in 68 cases (99%). The median size of cryobiopsies was 43.11 mm^2^ (range, 11.94–76.25). Diagnostic yield: among adequate TBLC the pathologists were confident (“high confidence”) that histopathologic criteria sufficient to define a specific pattern in 52 patients (76%), including 36 of 47 with UIP (77%) and 9 nonspecific interstitial pneumonia (6 fibrosing and 3 cellular), 2 desquamative interstitial pneumonia/respiratory bronchiolitis–interstitial lung disease, 1 organizing pneumonia, 1 eosinophilic pneumonia, 1 diffuse alveolar damage, 1 hypersensitivity pneumonitis and 1 follicular bronchiolitis. In 11 diagnoses of UIP the pathologists were less confident (“low confidence”). Agreement between pathologists in the detection of UIP was very good with a Kappa coefficient of 0.83 (95% CI, 0.69–0.97). Using the current consensus guidelines for clinical-radiologic-pathologic correlation 32% (20/63) of cases were classified as Idiopathic Pulmonary Fibrosis (IPF), 30% (19/63) as possible IPF, 25% (16/63) as other f-DPLDs and 13% (8/63) were unclassifiable.

**Conclusions:**

TBLC in the diagnosis of f-DPLD appears safe and feasible. TBLC has a good diagnostic yield in the clinical-radiological setting of f-DPLD without diagnostic HRCT features of usual interstitial pneumonia. Future studies should consider TBLC as a potential alternative to SLBx in f-DPLD.

## Introduction

Histology is a key element for the multidisciplinary diagnosis (MDD) of fibrotic diffuse parenchymal lung diseases (f-DPLD) when the clinical-radiological picture is nondiagnostic. Among f-DPLDs, idiopathic pulmonary fibrosis (IPF) is the most common and lethal form [1]. IPF is defined by the presence of usual interstitial pneumonia pattern (UIP) in the appropriate clinical context. In a half of patients, UIP pattern is detected radiologically (by high resolution computed tomography [HRCT]), in the remaining half of cases the diagnosis rests on histology obtained by surgical lung biopsy (SLBx) [2]. There are other conditions that may result in chronic interstitial fibrosis, including nonspecific interstitial pneumonia (NSIP), advanced sarcoidosis, chronic hypersensitivity pneumonia (HP), and fibrotic pulmonary Langerhans cell histiocytosis [3]. It is important to correctly diagnose these latter entities since they have a more favorable prognosis and response to treatment. SLBx is the most reliable method to histologically distinguish UIP from other forms of idiopathic interstitial pneumonias and other processes that may mimic IPF [1], [2]. However SLBx has appreciable costs and risks (including mortality: 2–6% within 90 days of the procedure) [4]–[7]. This provides a strong rationale to find alternative diagnostic tools for histopathologic diagnosis in this clinical-radiologic context of f-DPLD.

Recent studies have focused attention on the potential role of transbronchial biopsy (TBB) [8], [9]. In a retrospective controlled study [10] we reported a specificity and positive predictive value for TBB with regular forceps in the diagnosis of UIP of 96% and 93%, respectively. However the sensitivity and negative predictive value were low (30% and 50% respectively), in line with previous reports [8], [9]. The limited negative predictive value of TBB is related to a high proportion of inadequate and nondiagnostic fragments, mostly due to the small size of specimens, and associated artifacts (especially “crush” artifact) [8], [9], [11], [12]. To overcome these obstacles we have previously tested the use of larger forceps (Jumbo forceps) through the rigid bronchoscope under general anesthesia [13]. We learned how to control bleeding using the Fogarty catheter, and obtained more and larger specimens compared to conventional TBB, but still not comparable with the surgical samples. In recent years, a newer bronchoscopic biopsy method, the cryobiopsy, has demonstrated a superior diagnostic yield in comparison to conventional forceps biopsy [14], [15], [16]. The advantage of the cryoprobe is that larger pieces of tissue can be extracted with the freeze-thaw cycle. Preliminary data showed that this technique is safe and the occurrence of pneumothorax and bleeding are comparable to regular forceps biopsies. We hypothesized that TBLC may have an important role in the diagnostic evaluation of f-DPLD. Currently there are no studies addressing the feasibility and diagnostic yield of TBLC in this setting. Therefore, we designed this study in order to test the potential role of TBLC in the diagnosis of f-DPLD with nondiagnostic HRCT findings.

## Materials and Methods

This prospective study was approved by the Area Vasta Romagna Ethical Committee (Prot 2607/2010). Written informed consent for participation in the study was obtained from each patient before any study procedure. To prevent the risk of an unnecessary lung biopsy was finally decided to suggest surgical lung biopsy to the patients only when pathologists were not able to identify any sufficiently informative pathological pattern on TBLC. Seventy-three patients were enrolled into the study and sixty-three underwent TBLC between April 2011 and September 2012 at the Pulmonology Unit of GB Morgagni Hospital, Forlì, Italy. These patients had clinical and radiologic features of idiopathic f-DPLD, but insufficient elements to achieve a specific diagnosis of IPF or other ILDs according to current international consensus criteria [1], [2].

### Clinical assessment

We collected clinical data at the time of the first visit at our Institution. We extracted the following data: age, gender, past medical history, medications, smoking history, environmental exposure history, physical examination findings, laboratory results, and pulmonary function data. Patients had to be at least 18 years old, with a forced vital capacity (FVC) higher than 50% of predicted normal value, a diffusing capacity for carbon monoxide (DLco) higher than 35% of predicted normal value, and a pulmonary systolic arterial pressure estimated by echocardiography less than 40 mm Hg. Exclusion criteria included coagulopathy (platelet count <70,000×10^9^/L, prothrombin time international normalized ratio >1.5), FEV1 <0.8 L, diffuse bullous disease, hemodynamic instability and severe hypoxemia (PaO2 < 55 mm Hg on room air).

### Radiological assessment

HRCT images of 69 patients performed within 1 month prior to bronchoscopy were reviewed by the radiologist (SP). 1 or 1.5-mm collimation section were obtained at 10-mm intervals or volumetrically on multi-detector CT scanners with 0.6- or 1-mm collimation and 1-mm reconstruction. All images were reviewed at window settings optimized for lung parenchyma (width, 1500–1600 HU; level, −500 to −600 HU). All patients included in this study had a HRCT pattern of f-DPLD defined as “possible” UIP or “inconsistent” with UIP according to current guidelines (2). The radiologic pattern was also inconsistent with sarcoidosis, Langerhans cell histiocytosis or other patterns of cystic or nodular diseases.

### The procedure

A flexible cryoprobe measuring 90 cm in length and 2.4 mm in diameter was used (ERBE, Germany). The probe was cooled with carbon dioxide (CO2) which allowed the temperature in the probe's tip to decrease to −75°C within several seconds. The patients were deeply sedated with intravenous propofol and remifentanil and intubated with a rigid tracheoscope. The biopsies were obtained under fluoroscopic guidance using the flexible bronchoscope inserted through the rigid tube into the selected bronchus. Particular caution was given to the position of the biopsy: the cryoprobe was placed perpendicular to the chest wall to assure an accurate evaluation of the distance from the thoracic wall by fluoroscopy. A distance of approximately 10 mm from the thoracic wall was considered optimal. The biopsy site was decided by the bronchoscopist taking into consideration the HRCT scan appearance of each case. Once brought into position, the probe was cooled for approximately 5–6 s, then it was retracted with the frozen lung tissue being attached on the probe's tip. The frozen specimen was thawed in saline and then transferred to formalin for fixation. To minimize the consequence of hemorrhage, we performed each biopsy with a non-inflated Fogarty balloon placed in the lobar bronchus near the biopsy segment. The Fogarty balloon was always inflated after biopsy, then immediately deflated in case of absence of hemorrhage. In case of bleeding the Fogarty was deflated only after bleeding cessation, then we continued to perform additional biopsies. Within 3 h after the procedure, a chest X-ray was obtained to exclude pneumothorax. Oxygen was administered continuously through the rigid bronchoscope and spontaneous breathing was maintained during the procedure. Oxygen saturation, blood pressure, and ECG were monitored continuously.

### Pathologic assessment

**Figure 1:**
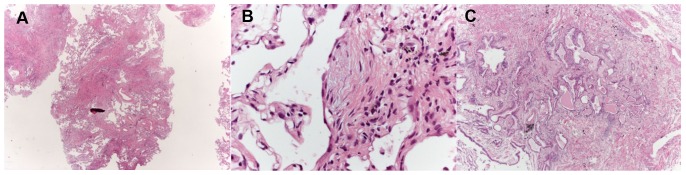
Different Examples of Cryobiopsy Showing UIP pattern. A) A low-magnification image showing dense scarring obliterating the alveolar architecture and abruptly alternating with relatively normal lung (patchy fibrosis). Some fibroblastic foci are visible even at this magnification for their pale-gray color. B) Fibroblastic focus better visualized at higher magnification. C) An area of honeycombing.

**Figure 2:**
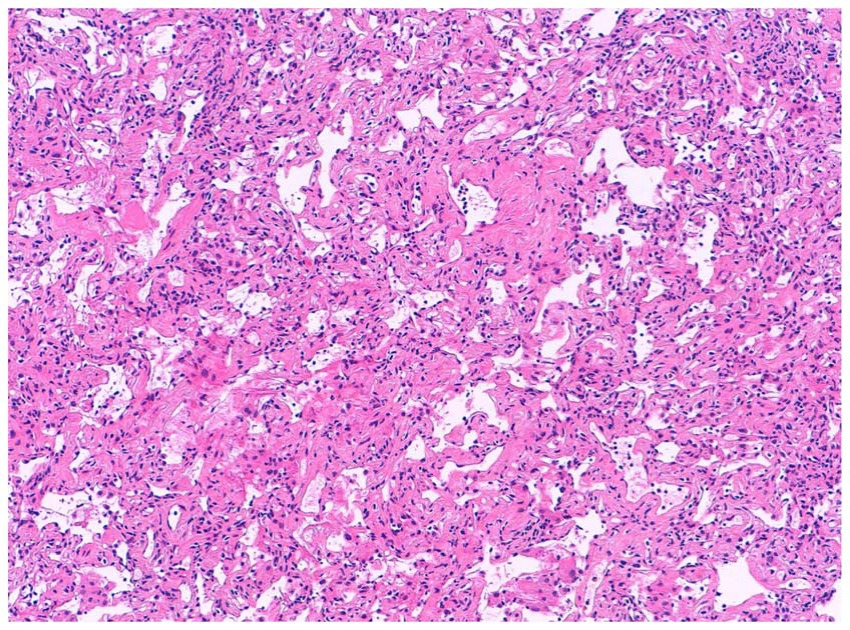
NSIP pattern. Lower lobe transbronchial cryobiopsy. A temporally homogeneous alveolar septal fibrosis is evident.

Hematoxylin-eosin–stained slides from TBLC were de-identified and sent to 2 pathologists (AC, TVC). The pathologists examined the specimens independently and in a blinded fashion; pathologic interpretations were made using the same features/criteria as identified on surgical biopsies and pathologists assigned a level of confidence (“high” or “low”) to their interpretations relative to confidence for similar diagnoses on SLBx. Divergent opinions were discussed and a consensus final diagnosis was reached in all cases. For analysis of the TBLC data the following parameters were recorded: the site where TBLC was performed, the number and surface area of TBLC pieces, the presence of pleural tissue and the presence of artifacts related to the procedure (“crush” artifacts and artifactual acute lung injury). At least one fragment of alveolated lung parenchyma was required to classify the biopsy as adequate. TBLC was considered nondiagnostic when histopathologic criteria sufficient to define a characteristic histopathologic pattern were lacking. Pathologic diagnosis of UIP was made according to current guidelines criteria [2] (Fig.1). All cases that exhibited pathological findings suggestive, or definitive for, an alternative diagnosis were considered non-UIP and classified according to the characteristic pathologic features, i.e. nonspecific interstitial pneumonia (NSIP) (Fig. 2), desquamative interstitial pneumonia (DIP), respiratory bronchiolitis-interstitial lung disease (RB-ILD), organizing pneumonia (OP), diffuse alveolar damage (DAD), hypersensitivity pneumonitis (HP), follicular bronchiolitis (FB), and eosinophilic pneumonia (EP).

### Statistical methods

Data were collected and analyzed after the final visit of the last patient enrolled. To evaluate the feasibility and diagnostic yield of TBLC in the diagnosis of f-DPLD we measured the yield of adequate biopsies and the yield of diagnostic biopsies. We evaluated TBLC in the detection of specific histopathologic features of f-DPLD including the histopathologic features of UIP and non-UIP patterns (i.e. NSIP, OP, DIP, RB-ILD, EP, DAD, HP, FB). The interobserver agreement for the final diagnosis between 2 pathologists was measured by K statistic and relative 95% confidence interval (95% CI). Safety analysis consisted in the evaluation of the incidence of severe (requiring medical intervention or hospitalization) and/or life threatening adverse events. Statistical analyses were carried out using SAS Statistical.

## Results

### Safety

None of the patients needed intervention to control bleeding, such as intubation with a double-lumen endotracheal tube or surgery. Despite the preventive use of Fogarty we observed prolonged bleeding in one case (1,4%). Pneumothorax occurred in 19 patients (28%) after the cryobiopsy procedure; and 14 (20% of total) required chest tube drainage. Median time of hospitalization after biopsy was 3 days (range, 0–9). In-hospital stay differs significantly between cases with and without pneumothorax, 6 days (range 1–9) and 1 day (range 0–1), respectively. The risk of pneumothorax was increased when fragments of pleura were present in the biopsy, but did not correlate with number or size of specimens (Table 1). One patient died (1.4% of the cases) seven days after biopsy of acute exacerbation of IPF, the coexistence of diffuse alveolar damage and UIP was confirmed at autopsy. The patient was HIV-positive and the procedure was complicated by massive pneumothorax which required the placement of drainage tube and treatment with high flow oxygen, which may have triggered the acute exacerbation.

**Table 1:** Prevalence and risk factors for Pneumothorax.

	WITH PNEUMOTHORAX	WITHOUT PNEUMOTHORAX	p
Cases, N (%)	19(28)	50(72)	
Nr. Fragments, median (range)	3(1–5)	3(1–6)	0,325
Total Area of Fragments in mm^2^, median (range)	47,48(14,06–73,92)	42,73(11,94–76,25)	0,146
Presence of Pleura, N (%)	11(58)	12(24)	0,008

Four patients were screened and enrolled in the study but did not undergo TBLC. The reasons were desaturation during the bronchoscopy in three cases and intercurrent atrial fibrillation before bronchoscopy in one case. It should be noted that all three patients that desaturated during rigid bronchoscopy were obese (BMI>30 Kg/m2). In these four cases only BAL was performed.

### Biopsy specimen characteristics

**Figure 3:**
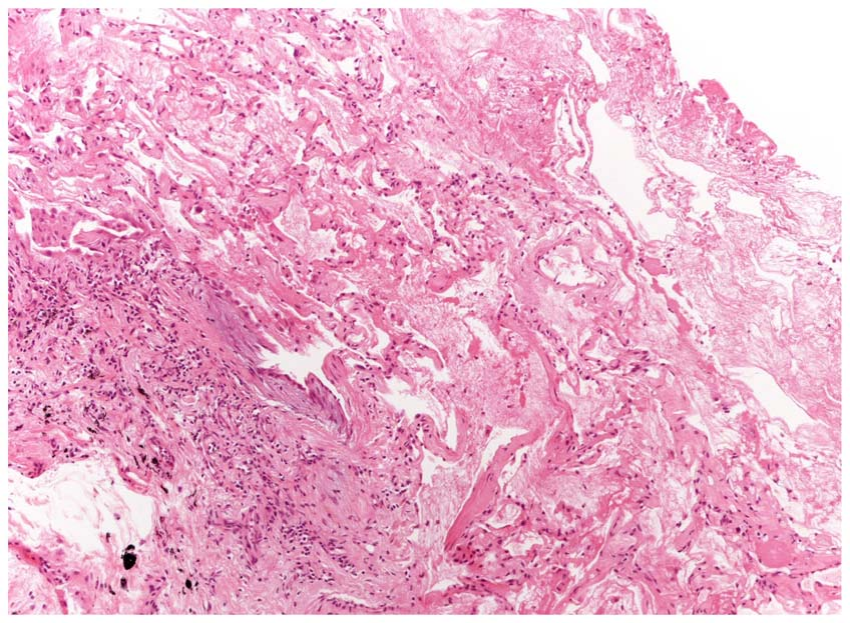
Freezing Artifacts. Artifactual acute lung injury probably related to tissue damage from cold consisting in edema, intra-alveolar fibrin and blood. The lesion is seen in the lower left corner, consisting in an area of patchy fibrosis with some chronic inflammation and a fibroblastic focus (UIP with high confidence).

67 (95%) TBLC were obtained in the lower lobes in only one segment. Median number of fragments was 3 (range, 1–6). Median size of fragments obtained for single patient was 43.11 mm2 (range, 11.94–76.25). All but one case demonstrated at least one fragment with alveolated lung parenchyma. Six biopsies were nondiagnostic; mean number of fragments was 2.6 (range 1–5). Between diagnostic and nondiagnostic biopsies there was not a statistically significant difference in terms of mean number of fragments, 3.19±0.96 and 2.67±1.37 respectively, p = 0.151. Interestingly, the area of fragments strongly correlates with the diagnostic yield, mean area was 41,99±14.73 mm^2^ for diagnostic cases and 28.43±11.66 mm^2^ for non-diagnostic cases, p = 0.038. Pleural tissue was detected in 23 biopsies (33%). Crush artifacts were not seen and the alveoli were not compressed (Fig. 1 and 2). Artifactual acute lung injury, probably related to tissue damage from cold and consisting of edema, intra-alveolar fibrin and blood, was detected in 40 (58%) cases. These artifacts were considered minimal in 25 cases (36%) while appeared more extensive in 15 (22%) cases (Fig. 3).

### Adequacy and diagnostic yield

**Figure 4:**
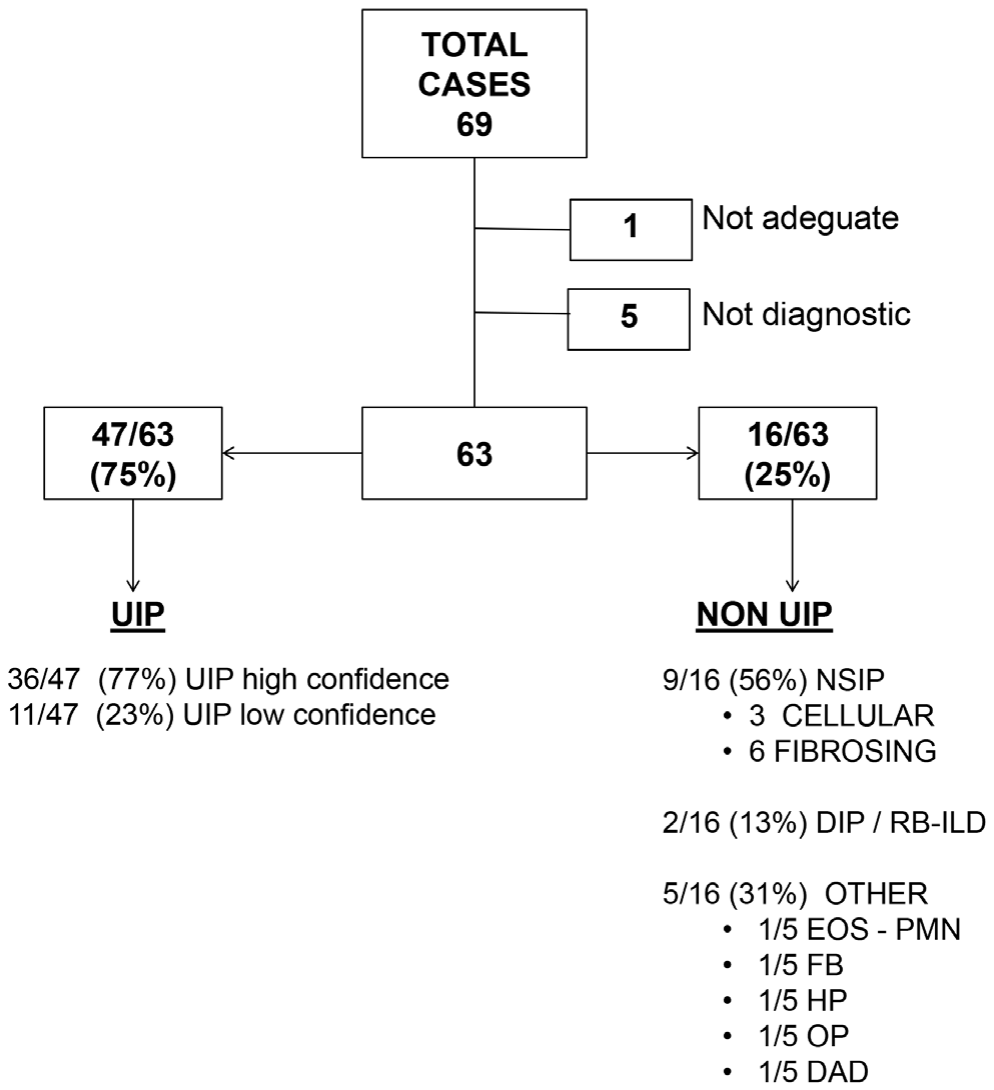
Description of Pathologic Interpretations. Abbreviations: EOS-PMN, Eosinophilic Pneumonia; FB, Follicular Bronchiolitis; HP, Hypersensitivity Pneumonitis; OP, Organizing pneumonia; DAD, diffuse alveolar damage.

Description of pathologic interpretations is reported in Fig. 4. Adequate cryobiopsies were obtained in 68 (99%) cases. Among adequate TBLC, the pathologists identified histopathologic criteria sufficient to define a characteristic pattern in 63 patients (93%), including 47 UIP (36 with high confidence and 11 with low confidence, Fig. 1), 9 NSIP (6 fibrosing and 3 cellular, Fig 2), 2 desquamative interstitial pneumonia/respiratory bronchiolitis–interstitial lung disease (DIP/RB-ILD), 1 organizing pneumonia (OP), 1 eosinophilic pneumonia (EP), 1 diffuse alveolar damage (DAD), 1 hypersensitivity pneumonitis (HP) and 1 follicular bronchiolitis (FB).

Cases of UIP showing a combination of well defined patchy fibrosis and fibroblastic foci, with or without honeycombing (Fig.1) typically were assigned a “high” confidence for the diagnosis; ie. all the features as seen in SLBx could be appreciated. Those cases of “UIP with low confidence” tended to show the following: patchy fibrosis + honeycombing (without fibroblastic foci), fibroblastic foci + honeycombing (without patchy fibrosis), or just one of the three main features of UIP (patchy fibrosis or fibroblastic foci or honeycombing); in most of these cases with low confidence of histologic diagnosis, the amount of tissue to evaluate (in comparison to SLBx) was the main problem. A detailed analysis of pathologic interpretations in UIP cases is reported in Table 2.

**Table 2:** Detailed Analysis of Pathologists' Interpretations of UIP cases.

	PATHOLOGIST 1	PATHOLOGIST 2
	N of Cases	N of Cases
**UIP-H (PF+FF)**	**27**	**31**
with HC	3	8
without HC	24	23
**UIP-L**	**19**	**16**
PF+HC	2	3
FF+HC	4	3
PF only	7	4
FF only	4	3
HC only	2	3

Abbreviations: UIP-H usual interstitial pneumonia-high confidence; UIP-L usual interstitial pneumonia-low confidence; PF patchy fibrosis; .FF fibroblastic foci; HC honeycomb lung.

### Controls: surgical biopsy and autopsy findings

Six diagnoses were confirmed by surgery or autopsy. Five diagnosis of UIP were confirmed by SLBx in three, autopsy in one case and lobectomy in one case (for coexistent lung cancer). One diagnosis of f-NSIP was confirmed by SLBx.

### Interobserver agreement between pathologists in the diagnosis of UIP

Interobserver agreement between the two pathologists for the recognition of UIP on TBLC was very good, with a Kappa coefficient of 0.83 (95% CI 0.69–0.97). Weighted Kappa coefficient of agreement for the identification of UIP with high or low confidence was good at 0,70 (95% CI 0,57–0,83). Analyzing the pathologic features of UIP separately, it appears that the agreement between the two pathologists is good with a Kappa coefficient of 0.64 (95% CI 0.43–0.87) for the recognition of honeycombing, 0.51 (95% CI 0.31–0.71) for fibroblast foci and 0.62 (95% CI 0.43–0.80) for patchy fibrosis.

The interobserver agreement between the two pathologists in the recognition of non-UIP patterns on TBLC was not measurable due to the limited number and different categories of other DPLDs evaluated in the study. In 11 (16%) cases the blind pathologic impressions were discordant and pathologists reached a final consensus diagnosis after discussion of cases. A description of discordant cases is reported in Table 3.

**Table 3:** Discordant Pathologic Blind Interpretations.

CASE	FINAL CONSENSUS	BLIND ANALYSIS	
		Pathologist 1	Pathologist 2
1	UIP-L	chronic HP	UIP-L
2	f-NSIP	UIP-L	f-NSIP
3	c-NSIP	chronic HP	c-NSIP
4	c-NSIP	c-NSIP	ND
5	OP	OP	c-NSIP
6	*EOS-PMN*	*EOS-PMN*	OP
7	DAD	OP	DAD
8	UIP-L	f-NSIP	UIP-L
9	ND	UIP-L	fibrotic OP
10	UIP-H	ND	UIP-H
11	c-NSIP	ND	c-NSIP

Abbreviations: UIP-L usual interstitial pneumonia-loww confidence level; UIP-H usual interstitial pneumonia-high confidence level; c-NSIP, cellular non specific interstitial pneumonia; f-NSIP, fibrotic non specific interstitial pneumonia EOS-PMN, Eosinophilic Pneumonia; HP, Hypersensitivity Pneumonitis; OP, Organizing pneumonia; DAD, diffuse alveolar damage; ND, non diagnostic.

### Patient characteristics and IPF diagnosis

**Figure 5:**
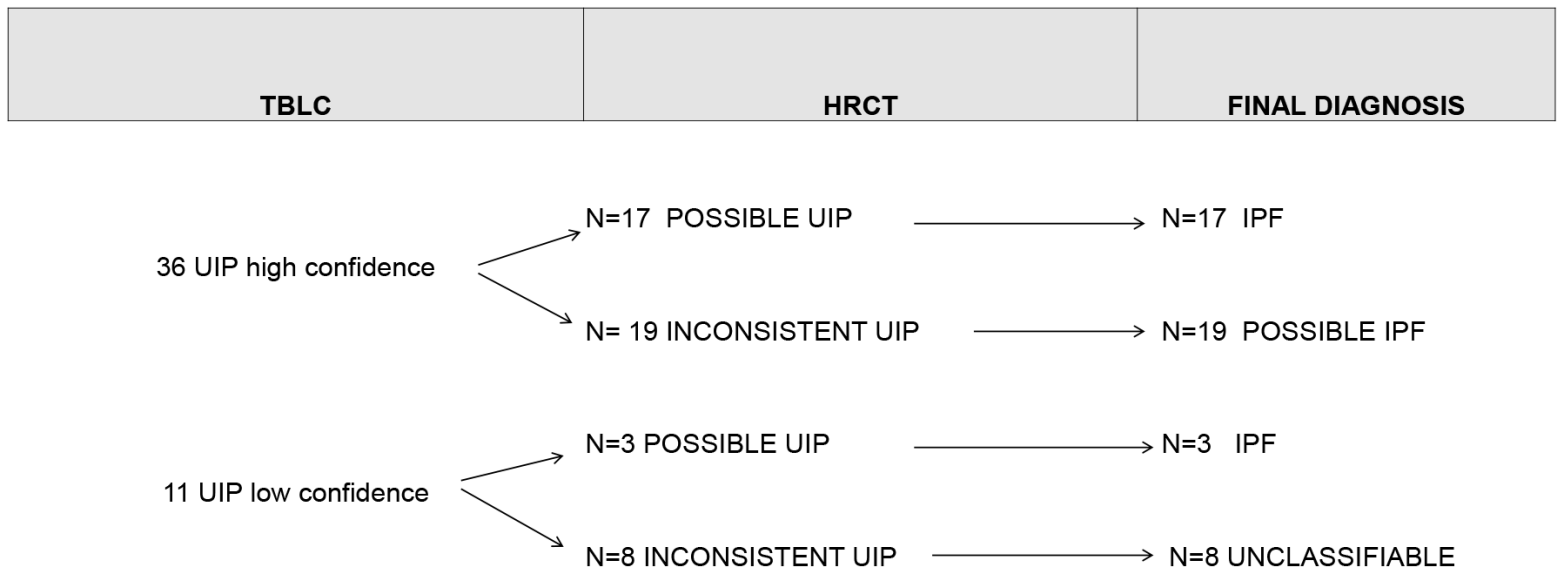
Correlations between Pathologic Interpretations of UIP cases and Final Diagnosis. Report of pathologic interpretations and final diagnosis of 47 cases with UIP histologic features on cryobiopsy. TBLC column shows pathologic interpretations. HRCT column report radiologic characteristics of cases based on high resolution tomography findings. Last column summarises the final diagnosis achieved according to current ATS/ERS criteria.

Patient characteristics are reported in Table 4. Using the criteria in current consensus guidelines (2), among 47 cases in which pathologists detected some or all features of UIP, 20 were classifiable as IPF and 19 as possible IPF, while the remaining 8 cases were unclassifiable (Fig. 5).

**Table 4:** Patients characteristics (Nr total  = 69 pts).

		Nr(%)
**HRTC**	Possible UIP, N (%)	22(32)
	Inconsistent UIP, N (%)	47(68)
**Gender**	Male, N (%)	35(51)
**Smoking History**	Never, N (%)	29(42)
	Former, N (%)	35(51)
	Current, N (%)	5(7)
	PY, median (range)	20(3–60)
**Age in years, median (range)**		60(29–77)
**FVC % of predicted, median (range)**		81(50–23)
**DLCO % of predicted, median (range)**		55(29–86)

## Discussion

TBLC in the diagnosis of f-DPLD appears safe and feasible. TBLC has a good diagnostic yield in fibrosing interstitial pneumonia in the clinical-radiological setting of f-DPLD when high resolution computed tomography (HRCT) features diagnostic of usual interstitial pneumonia (UIP) are not present. Pathologists are generally confident in their ability to make diagnoses using essentially the same criteria as for SLBx.

Safety is one important concern. Rigid bronchoscopy under deep sedation was feasible in 94,5% of screened patients. Obesity (BMI > 30 Kg/m2) was related to oxygen desaturation during deep sedation, excluding the possibility to perform TBLC in 4% of cases. Our pilot study, for safety concerns, was designed to hospitalize and carefully observe all patients, thus probably over-estimates the hospital length of stay. However even under this unfavorable scenario our data shows that the time of hospitalization is shorter than length of stay in our institution after SLBx, that is 4.8 days (range,3–9). Reported median time of hospitalization after SLBx procedure for any cause ranges between 4 to 8 days [17]–[18], but elderly patients with ILDs are at higher risk of prolonged hospitalization (more than 7 days) [19]. Given our preliminary results future studies might reasonably propose TBLC in an outpatient setting with hospitalization only of selected cases, particularly when complications occur. Bleeding and pneumothorax represent the most frequent complications of transbronchial biopsy. The preventive use of Fogarty does not allow us to quantify the true incidence of bleeding complications, but prolonged bleeding occurred in one case (1,4%). Based on our experience we recommend the preventive use of Fogarty catheter in all cases. We screened patients by echocardiography to exclude cases with pulmonary hypertension, this might have helped in preventing severe bleeding. However, whether the routinely use of echocardiography before TBLC should be recommended is beyond the objective of this study and remains to be addressed in future trials. Previous studies of TBLC reported the occurrence of pneumothorax in 5% of cases, comparable to regular forceps biopsies or even lower in a subset of patients under surveillance for lung transplant [14]–[16]. In our study pneumothorax occurred in one-third of cases and the presence of pleura in the biopsy was associated with increased risk. We did not find a correlation between the incidence of pneumothorax and the number or size of the biopsies. The higher incidence of pneumothorax may be explained by the characteristics of our study population limited to fibrosing DPLD. This hypothesis is in line with our previous results showing that the percentage of pneumothorax by TBB in f-DPLD was higher than for other DPLDs (8% of cases) [10]. The striking incidence of pneumothorax is mainly related to the necessity of biopsy the subpleural areas in this selected group of patients with fibrotic ILDs. HRCT is essential to choose the site to biopsy in these diseases, but the use of HRCT does not lower the incidence of pneumothorax. The decision of taking peripheral subpleural biopsies in this setting is forced by the exigency of obtaining diagnostic samples that cannot be obtained with more central biopsies. Acute exacerbation of IPF caused the death of one patient 7 days after the procedure. This is the first report of a case of death after cryobiopsy in DPLD. Death from acute exacerbation after transbronchial biopsy can occur, but is infrequent, 0.3% among 320 patients evaluated for IPF in our center (unpublished data). Reported mortality for surgical lung biopsy is higher (2–6%) [4]–[7], and in our Institution is 3,9% (unpublished data). These preliminary results, show that the technique appears to be safer than surgical lung biopsy, but not harmless. Among 113 cases collected to date at our centre, one died (0,9%) and this lead us to the conclusion that before this tool is widely adopted we need to test its safety in larger multicentric trials.

The lung specimens collected in this study were large and well preserved. Almost all specimens were adequate and the quantity of tissue sampled was sufficient in the majority of cases to reach a pathologic interpretation of cases. Pathologists were able to reach a diagnosis even in cases with only one fragment. The number of biopsies does not correlate with the diagnostic yield, but the size of specimen does. We observed a higher diagnostic yield in cases with larger total area of fragments. Crush artifacts were not observed with cryobiopsy, whereas they are nearly always present in specimens obtained by forceps biopsy [20]–[24]. In cryobiopsies, acute lung injury changes (probably procedure-related) were frequently present, but they were generally recognized as artifacts and did not significantly interfere with pathologists' interpretations. Comparing the data of this study to our previously published results on regular forceps TBB in UIP, performed on a similar population (f-DPLD with non diagnostic clinical/radiological findings) and evaluated by the same 2 pathologists [10], it appears that the proportion of diagnostic biopsies is dramatically increased, from 30% for TBB to 93% for TBLC. TBLC significantly improve the diagnostic yield of TBB for detection of UIP. Our results show that TBLC in patients with f-DPLD can identify UIP with high confidence level in 52% of cases and with low confidence level in 16% of cases. In our previous study we reported that TBB in UIP cases detected all three features of UIP in only 0–2.5% of cases, two features in 10–22.5% of cases and one feature in 5–20% [10].

Although this study was not specifically designed to evaluate the impact of TBLC in the MDD of cases, TBLC yields a diagnosis of definite IPF in one third of cases, and possible IPF in another third of cases using the diagnostic criteria of current consensus guidelines. The current IPF guidelines provide a framework for assessing radiologic and pathologic features in suspected cases of IPF. The terms “definite, probable, possible, and not UIP” are used for the pathologic findings. While it is tempting to apply those terms to cases in our series, we do not think it is appropriate since those terms refer to features in SLBx. Our subjective assessment of confidence of pathologic interpretation of TBLC should in no way be equated with those terms.The agreement between pathologists for the recognition of UIP pattern was very good and significantly higher compared to what we previously reported for TBB (kappa 0.83 for TBLC compared to 0.61 for TBB). Previous studies documented similar coefficient of agreement between pathologists in the diagnosis of UIP also for SLBx [25], [26], [10]. However we acknowledge that this study was not compared to surgical lung biopsy and we cannot exclude the possibility that the impressive agreement between pathologists reflects a bias related to the smaller quantity of material and pathologic findings of cryobiopsy in which we might miss important ancillary information. Against this criticism the fact that in all 6 cases in which patients gave their consent, we had a surgical confirmation of diagnosis. Compared to regular forceps biopsy, cryobiopsy improves pathologists' agreement for the detection of patchy fibrosis (Kappa 0.62 for TBLC compared to 0.29 for TBB), whereas no change in the agreement on the recognition of honeycombing (Kappa 0.64 for both TBLC and TBB) and fibroblast foci (Kappa 0.50 for TBLC compared to 0.51 for TBB) was observed. Nicholson et al. [25] reported levels of agreement between pathologists for individual histological criteria of UIP in SLBx to be higher than TBLC (mean K 0.75 for fibroblast foci and 0.76 for honeycombing). Documenting the presence of patchy fibrosis with regular forceps TBB is difficult for the small size of samples and for the presence of crushing artefacts. It is not surprising that with TBLC and SLBx this specific feature of UIP is better recognized by pathologists compared to TBB.

A potential bias in this study is the fact that most cases had UIP and the usefulness of TBLC in other forms of ILD may not be generalizable. The three most challenging differential diagnoses were NSIP, UIP and chronic HP, representing the majority of discordant pathologic diagnosis in the blind analysis. Also for SLBx, distinguish UIP from other diseases such as DIP, NSIP, chronic HP can be rather challenging [25]–[27]. Nevertheless SLBx performed in two lobes can detect cases in which different pattern coexist and we recognize in the single lobe biopsies performed in this study and in the lack of reliable controls (SLBx) two relevant limitations.

We acknowledge that the absence of SLBx limited the possibility to compare the accuracy of TBLC in terms of sensitivity and specificity to SLBx for the diagnosis of f-DPLD. However, we believe that a prospective randomized trial of cryobiopsy versus SLBx is not ethical and practical in this era. Moreover, the blinded pathologic review of the single lobe biopsy used for the purpose of this study does not allow us to know how TBLC behaves when two lobes may be biopsied and clinical-radiological and bronchoalveolar lavage (BAL) data are discussed with the pathologists [25], [28]. For future studies, we propose to assess the role of cryobiopsy as a potential replacement for SLBx in the MDD process. However, given the lack of data on safety and feasibility of this technique in f-DPLD, we think that these preliminary results provide cardinal insights. Based on these results, larger and multi-center trials evaluating the impact of TBLC on the final MDD of f-DPLD are advisable for the future.

In summary, we conclude that TBLC in the diagnosis of fibrosing interstitial pneumonia in the clinical-radiological setting of f-DPLD with nondiagnostic high resolution computed tomography (HRCT) features appears safe, feasible and has a good diagnostic yield. Future studies should consider the use of TBLC as a potential alternative to SLBx in f-DPLD.
